# Investigation of Drinking Water Quality in Kosovo 

**DOI:** 10.1155/2013/374954

**Published:** 2013-02-21

**Authors:** Fatlume Berisha, Walter Goessler

**Affiliations:** University of Graz, Stremayrgasse 16, 8010 Graz, Austria

## Abstract

In the recent years, not much environmental monitoring has been conducted in the territory of Kosovo. This study represents the first comprehensive monitoring of the drinking water situation throughout most of the territory of Kosovo. We present the distribution of major and minor trace elements in drinking water samples from Kosovo. During our study we collected 951 samples from four different sources: private-bored wells; naturally flowing artesian water; pumped-drilled wells; and public water sources (tap water). The randomly selected drinking water samples were investigated by routine water analyses using inductively coupled plasma mass spectrometry (ICPMS) for 32 elements (Li, Be, B, Na, Mg, Al, K, Ca, V, Cr, Mn, Fe, Co, Ni, Cu, Zn, Ga, As, Rb, Sr, Mo, Ag, Cd, Sn, Sb, Te, Ba, Tl, Pb, Bi, Th, U). Even though there are set guidelines for elemental exposure in drinking water worldwide, in developing countries, such as Kosovo, the lack of monitoring drinking water continues to be an important health concern. This study reports the concentrations of major and minor elements in the drinking water in Kosovo. Additionally, we show the variation of the metal concentration within different sources. Of the 15 regulated elements, the following five elements: Mn, Fe, Al, Ni, As, and U were the elements which most often exceeded the guidelines set by the EU and/or WHO.

## 1. Introduction

The importance of water and its impact on human health and the environment resulted in the establishment of the Water Framework Directive (WFD) by the European Union (EU). The goal of WFD is to achieve qualitative and quantitative status of all water bodies by 2015 in all EU member states [1]. While European member states continue to control the sources of pollution and focus on improving the quality of the environment, developing countries, such as Kosovo, lack basic monitoring of any kind of water pollutants, and, as a consequence, human health could be at risk. 

Kosovo declared independence on the February 17, 2008. The youngest European country is still far behind the central European standards. Before the war of 1999, one of the Kosovo's mining sites, “Trepça,” with its 40 mines, millings, smelters, and factories, was considered to be one of the most important mining districts in Europe [2]. The site production has now ceased but no information on the risk of metal exposure or how the mining sites deposited their wastes throughout the territory of Kosovo is available. Smelters, mines, and industrial activities lead to metal contamination of soil, which can influence the quality of groundwater [2]. More than a decade later, there are still no studies on the environmental pollution or its impact on the human population. Groundwater movement is quite slow; therefore, even pollution deposited years earlier from industrial, agricultural, or even construction activities will impact human years later [3, 4]. It is well known that various elements occur naturally in the groundwater in many parts of the world and many people are ignorantly exposed to them. The lack of research and modern analytical facilities is of major concern for public health. Furthermore, Kosovo has limited water resources which in the future will be a limiting factor for economic and social development. Any new scientific findings and resources will be useful to Kosovo's authorities and researchers as they plan and construct future water monitoring institutions to provide efficient protective measures for its own population, as well as neighboring populations. An attempt to study the groundwater profile in Kosovo has not yet been conducted. In addition, aside from its own population, Kosovo's water quality could have a negative effect on the neighboring countries, especially considering that the drainages of the Kosovo's main four river basins are outside of Kosovo's territory: “Drini i Bardhe” drains into the Adriatic Sea across Albania; “Ibar” and “Morava e Binqes” drains into the Black Sea across the Danube River through Serbia; and “Lepenci” drains into the Aegean Sea across Vardar in Macedonia [4, 5], which means that no polluted water crosses into Kosovo's territory. Moreover, the Organization for Security and Co-operation in Europe (OSCE) Mission in Kosovo in its 2008 report on human rights reported water shortages, poor water infrastructure, and contamination of water sources especially in rural areas. The report questioned the wells' and springs' quality as well as their impact on human health [5].

The European Union and World Health Organization (WHO) have set guidelines of drinking water quality [6, 7]. The objective of the drinking water guidelines is to protect the health of the consumers. Developing countries, such as Kosovo, need to comply with the EU and WHO guidelines for regulation and set standards for safe drinking water.

To date, most of the articles published about the environmental state of Kosovo are focused on depleted uranium [4, 8–15] with few soil science studies [2, 16, 17]. Information on drinking water and/or groundwater streams is limited. This led us to explore the drinking water and/or groundwater quality in Kosovo by analyzing the elemental composition of drinking water samples using inductively coupled plasma mass spectrometry (ICPMS). ICPMS is a suitable analytical technique for environmental trace analysis with low detection limits, multielemental qualifications, and high sample throughput [18]. Therefore, we present the first picture of the drinking water profile in Kosovo and whether the quality meets EU and WHO drinking water standard guidelines. We measured 32 elements (Li, Be, B, Na, Mg, Al, K, Ca, V, Cr, Mn, Fe, Co, Ni, Cu, Zn, Ga, As, Rb, Sr, Mo, Ag, Cd, Sn, Sb, Te, Ba, Tl, Pb, Bi, Th, U), of which fifteen elements are regulated by the EU or WHO. Additionally, we will discuss and compare the quality of different water sources.

## 2. Materials and Methods

### 2.1. Study Site Description

Kosovo has a land size of 10,887 km^2^ (between 42°41′56.85′′N and 21°11′18.92′′E and 42°39′59.20′′N and 20°26′59.26′′E), a population of approximately 2 million, and a density of about 200 people per km^2^. The land is mostly used for agriculture and forestry with little industrial activities. Kosovo is located in the middle of southern Europe, in an area known as the Balkans, and is bordered by Macedonia, Albania, Montenegro, and Serbia. The geographical location of Kosovo connects central and southern Europe, as well as the Adriatic Sea and the Black Sea. It is quite a strategically important region.

**Figure 1:**
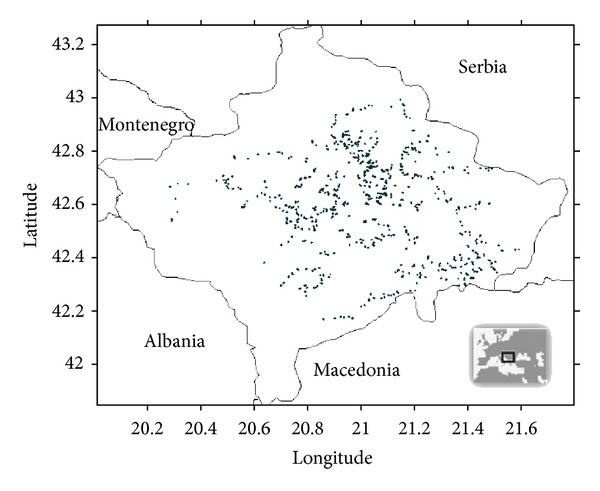
Map of Kosovo and location of the sample sites.

Throughout Kosovo, Figure 1, 951 drinking water samples were collected from privately bored wells, naturally flowing artesian water, pumped drilled wells, and public water sources (tap water). The majority of the samples taken are from private, individually bored wells coming from groundwater. These wells represent the primary water supply for most of Kosovo's population. The water-sampling points were chosen randomly, mostly in the rural areas and based on the population density of the country. Information about the sampling points was collected on an individual basis for every drinking water source during sampling and recorded in the field logbook. 

The sampling coordinate points were obtained with Google Earth, version 5, and recorded in the field logbook, whereas the drawing of the Kosovo maps, Surfer, version 8.00 (Golden Software Inc., CO, USA) was used.

### 2.2. Reagents and Reference Materials

Samples in the field were acidified with Trace Metal Grade 67–70% nitric acid (Fisher Scientific, Germany). The solutions and reference materials were prepared with Milli-Q water purification system with a specific resistivity of 18.2 MΩ cm (Millipore, Milford, MA, USA). Concentrated nitric acid (p.a) from Merck (Darmstadt, Germany) was further distilled in a quartz subboiling distillation unit. Stock standard solutions MerckVI multielemental (Merck, Darmstadt, Germany) and Communications and Power Industries (CPI) International (Santa Rosa, CA, USA) were used for preparation of external and internal calibration standards. Commercially available reference water materials form National Institute of Standards and Technology (NIST) Standard Reference Materials (SRM), NIST SRM 1643e (Gaithersburg, MD, USA), and Department for Agrobiotechnology (IFA) Test Systems (BOKU, Tulln, Austria) reference water with certified elemental concentrations were used for analytical quality controls.

### 2.3. Sample Collection and Preparation

The water samples were collected between June, 2009, and March, 2010, in 50 mL polypropylene sterile cellstar centrifuge tubes (Greiner Bio-One, Frickenhausen, Germany). For each sampling point, the same sampling team collected the samples. Before sampling, the water was run for at least 5 minutes, and the vials were rinsed three times with the water to be sampled. The vials were first filled with 2/3 of the maximum volume, closed with the caps and shaken for approximately 30 seconds. The rinsing water was discarded and the vials were filled with the water to the upper edge. Replicates of every sample were collected as a backup. The replicate samples were not used for analysis. In the field, 100 *μ*L trace metal grade 67–70% nitric acid was added. The water samples were labeled with a unique sample identification number and packed for transport to Graz for analysis. All of the samples were analyzed approximately one month after sampling. The samples were analyzed in groups. 

Water samples were neither filtered nor diluted prior to elemental measurement. They were transferred to 10 mL polystyrene single use tubes (Brand, Wertheim, Germany) and acidified to 10% with nitric acid (v/v). Studies have shown that the discrepancy between filtered and unfiltered samples is quite small and that filtration could in fact introduce contamination of samples [19]. Furthermore, we wanted to have the exact information on the quality of the drinking water distributed to people of Kosovo. 

All calibration standard solutions and blank solutions were prepared with ultrapure water from the Milli-Q purification system in a 10 mL polystyrene tube. Three different calibration standard solutions were prepared daily in order to cover the wide range of elemental concentration in the drinking water. The first set of calibration standards with a concentration range of 0.01 to 1 *μ*g/L, consisting of elements Li, Be, B, Al, V, Cr, Mn, Fe, Co, Ni, Ga, As, Cu, Zn, Rb, Mo, Ag, Cd, Sn, Sb, Te, Ba, Tl, Pb, Bi, Th, U, was diluted from 1000 mg/L Merck VI multielemental stock standard solution and 1000 mg/L, Sb, Sn, Th, respectively, from CPI stock standard solutions. The second set of calibration standards of concentration ranging from 5 to 100 *μ*g/L was diluted from 1000 mg/L Merck VI multielemental standard solution of Li, Be, B, Al, V, Cr, Mn, Fe, Co, Ni, Ga, As, Cu, Zn, Rb, Mb, Ag, Cd, Te, Ba, Tl, Pb, Bi, U. The third calibration set of standard solutions with a concentration range from 50 to 50000 *μ*g/L of Na, Mg, K, Ca, Sr was prepared from 10000 mg/L standard solutions of Na, Mg, K, Ca, and 1000 mg/L Sr from CPI stock standard solution, respectively. The calibration standard solutions were acidified to 10% (v/v) with nitric acid. Finally, the internal standards of 500 *μ*g/L of Sc, Ge, In, Lu, were prepared from 1000 mg/L CPI stock standard solutions. 

### 2.4. ICPMS

The routine analyses of the water samples were performed using inductively coupled plasma mass spectrometry (ICPMS, Agilent 7500ce, Agilent Technologies, Waldbronn, Germany). The ICPMS was equipped with a collision/reaction cell system, an ASX-510 autosampler (CETAC, Nebraska, USA), an integrated sample introduction system (ISIS) and a Mira Mist nebulizer (Burgener Research Inc, Ontario, Canada). The following isotopes were measured in the no-gas mode (^7^Li, ^9^Be, ^11^B, ^43^Ca, ^65^Cu, ^66^Zn, ^85^Rb, ^88^Sr, ^95^Mo, ^105^Ag, ^111^Cd, ^118^Sn, ^121^Sb, ^125^Te, ^137^Ba, ^205^Tl, ^208^Pb, ^209^Bi, ^232^Th, ^238^U) and Helium mode (^23^Na, ^24^Mg, ^27^Al, ^39^K, ^51^V, ^53^Cr, ^55^Mn, ^56^Fe, ^59^Co, ^60^Ni, ^71^Ga, ^75^As). Whereas, ^45^Sc, ^74^Ge, ^115^In, ^175^Lu were used as online internal standards for corrections. 

### 2.5. Measurement and Analysis

When analyzing a large number of samples as well as a wide range of elemental compositions, it is quite important to prove that the analytical method used in the study is acceptable for its intended purpose. To do so, we performed daily instrumental optimization and used online internal standard. We ensured precise measurements through reference materials for the trueness of our measurements and through the drift control and repeatable sample measurements.

The plasma operating conditions such as torch alignment, RF power, and nebulizer flow rate were selected for high sensitivity and low oxide ratio. Additionally, suitable ion lens voltage for maximal signal was optimized daily with the tuning solution (1 *μ*g/L Li, Y, Tl, Ce, Co, Fe, Se, and Cr in 2% HNO_3_). To maximize the signal, reduce matrix effect, and balance the measurement drift, the internal standard solution of Sc, Ge, In, and Lu, covering the range of all atomic masses of the elements analyzed, was continuously nebulized online with the final concentration of 100 *μ*g/L.

To ensure accurate measurements of trace elemental analysis of the drinking water samples, NIST SRM 1643e and IFA Test Systems reference water were analyzed at regular intervals. When measuring a wide range of elements, it is hard to find a suitable reference material for all of the elements analyzed. To further ensure precise analytical methods throughout of the entire sample analysis, a blank, a drift control and duplicate sample where measured repeatedly after every 15th sample. The concentration of the duplicate samples was nearly identical and, moreover, drift stability over a period of 28 hours was achieved.

From the pool of 951 samples, we resampled 32 samples with higher and unrealistic concentrations of certain elements. The comparison of the results between the repeated samples showed good agreement. 

The elemental concentration of most of the samples was above the calculated quantification limit, where major elements had detection limits in the *μ*g/L scale depending on the element, whereas for the trace elements the detection limit was in ng/L range. We present elemental concentrations only above the smallest concentration of our lowest measured calibration standards (0.01 *μ*g/L). 

Statistically, the data was handled by Stata data analysis and statistical software, version 7.0 (Stata Corporation, TX, USA) and Excel 2007. A statistically significant (*P* = 0.05) difference of drinking water sources for the different elemental concentrations measured was tested. 

Due to the fact that a high percentage of samples was below the limit of quantification (Be 87%, Ga 70%, Ag 96%, Sn 73%, Te 71%, Tl 84%, Bi 95%, and Th 83%) only 23 out of the 32 elements analyzed will be reported and discussed here. Mercury requires preservation, which was not performed during the field sampling in Kosovo and as a result will not be reported or discussed. Furthermore, for the sake of brevity, U will be discussed elsewhere. 

## 3. Results and Discussion

The quality of drinking water is a worldwide concern. It is important to monitor its quality prior to any further precautions [20]. This study presents and reports the concentration levels of metals in the drinking water and/or groundwater samples by analyzing the four different sources of water samples from Kosovo. 

Trace elemental concentration of NIST 1643e SRM reference water solution is often used to validate the analytical techniques during measurements. The quality control results of the certified and measured values of NIST 1643e SRM reference water, *n* = 7, are presented in Table 1. The measured values agree well with the certified values. 

The main statistical parameters (mean, min, max, median, and 25th and 75th percentile) of the elemental concentrations (*μ*g/L) for the four sources of drinking water are summarized in Table 2. Table 2 also shows the percentage ≤ LOQ as well as the EU and/or WHO limit of drinking water guidelines. From 951 samples, 68.0% are private-bored well samples, 21.1%, naturally flowing artesian water, 7.4% pumped-drilled well, and 3.5% from public water source. Private-bored well drinking water sources are the main water supplier for the majority of the Kosovo's population and therefore it needs greater attention. 

The drinking water analyzed was compared to the limits and recommendations of the EU and WHO drinking water standards (Table 2).  Table 3 presents the number of samples that surpass the levels of EU and WHO guidelines in drinking water, respectively. It can be clearly ascertained that the samples exceeding the limits were mostly from the private-bored wells, then naturally flowing artesian water, pumped-drilled well, and public water source, respectively. Of the 14 (U excluded) regulated elements by either EU and/or WHO, the elements that surpassed the set levels most often, when compared to the total samples for that element, were Mn, Fe, Al, Ni, and As. 

From Table 2 it can be clearly determined that there is a large variation for the elemental concentrations and a similar situation for elemental concentrations of different sources. Moreover, for most of the minor elements analyzed, we encountered a wide range (2 to 4 orders of magnitude) of elemental concentrations. This span is not as evident for the major elements (Na, Mg, K, Ca), yet the median concentration differs between the sources for the major elements and some minor elements, respectively (Table 2). Mn mean concentrations vary significantly between the sources, with the highest concentrations in the private-bored wells. Similar patterns are seen for Fe, Cr, Co, Zn, Cd, and Sb. These variations cannot be explained due to the lack of geological and environmental studies in Kosovo. However, one can postulate that the elements that show higher orders of magnitude could have no solubility controls if present in an oxidizing environment; they could be redox and pH sensitive or they could have various concentrations in rocks [21, 22]. Further, with ANOVA analysis we tested the source variation on whether the means are equal. Hence, we determined that the concentrations between groups are significantly different for the following elements: Li, Na, Mg, K, Ca, As, Rb, Sr, Mo, Sb, and Ba. Additionally, we tested the differences in variance between different sources of water using Bartlett's test for equal variances with *P* value less than 0.05 showing that the variances are not the same across groups. In this case, we determined that the only element with the same variance across different sources of water was Mg. 

In our study we found 792 samples to exceed at least one or more elements of either EU and/or WHO drinking water concentration guidelines. These samples are not significantly different between the sources. If we set the criteria to consider four or more elements surpassing any of the guidelines, we found only 57 sample points exceeding the set concentration guidelines, from which we can note that most of these sample points are private-bored well (43.9%), followed by naturally flowing artesian water (38.6%), pumped-drilled well (10.5%), and public water source (7.0%), respectively. Private-bored well drinking water source dominates within the highest number of samples surpassing the concentration guidelines. 

**Figure 2:**
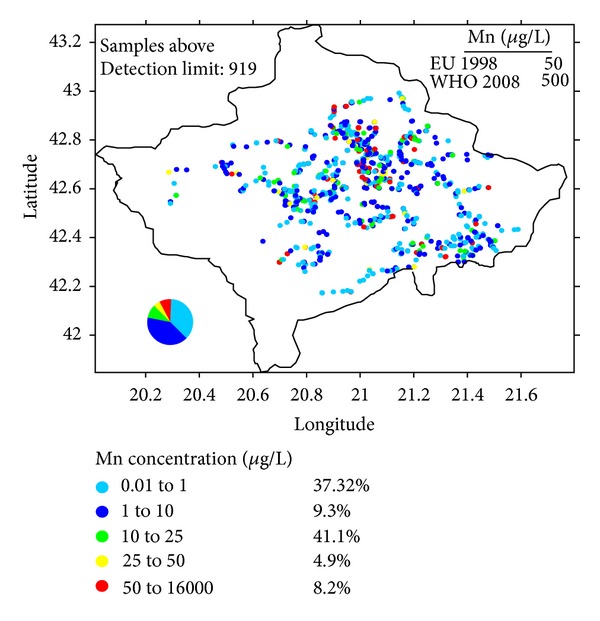
The distribution of Mn concentration.

We also performed some linear regression analysis testing the effect of depth (1–150 m) and age (up to 250 years) of the water source and latitude and longitude on the risk of the drinking water. Neither the depth nor the age showed a correlation with the concentration of the contaminants. After elimination of the variables with the highest *P* values we found the latitude and longitude as significant variables. Regression analysis showed that as the latitude of the water source increased, the risk for higher elemental concentration in the water increased. A similar regression analysis showed a similar pattern with longitude: an increase in longitude of the water source showed an increase in the risk for higher elemental concentration. This implies that the northeast area of Kosovo should have a higher number of contaminated samples. In Figure 2, we show the distribution of the Mn concentration. 

### 3.1. Health Implications

The importance and the health implications of selected elements are discussed. Particular attention is given to elements exceeding the EU and WHO guidelines, yet some unregulated elements with noted health implications are also discussed. 

Previously, we singled out Mn, Fe, Ni, As, and Al as the elements surpassing the EU and WHO guidelines in the largest number of samples tested. According to WHO, when high concentrations of Mn and Fe are observed, the iron bacteria may cause deposits in the drinking water source and, therefore, may compromise the acceptability of the drinking water [7].

Manganese (Mn) is an essential element in small quantities mainly for bone development and the metabolism of amino acids, lipids, and carbohydrates. In excess amounts, however, Mn has been shown to be toxic causing hyperactive behavior in infants and neurotoxin effects. Additionally, areas with a high concentration of Mn in drinking water report higher infant mortality [23]. In the brain, high doses of Mn can cause Parkinson syndrome [22, 24]. A high concentration of Mn in drinking water also causes an unpleasant taste [22, 25]. The moderate presence of manganese in drinking water has no direct health effects, but the precipitated manganese in water can lead to aesthetic and infrastructure problems. A precipitate of manganese is usually black [22]. In our analyses, a total of 74 samples exceeded the drinking water limit of 50 *μ*g/L set by the EU and only 20 samples exceeded the limit of 400 *μ*g/L set by WHO. The highest concentrations of Mn were observed in private wells.

Iron (Fe) is a necessary element for the biological functions of the human body, yet in excess can lead to acute Fe poisoning [21]. The EU guideline for Fe in drinking water is 200 *μ*g/L, which was exceeded by 62 samples.

Nickel (Ni) is an essential element in small quantities, yet in high concentrations can cause heart and liver damage and skin irritation [21, 26]. The EU guideline for Ni in drinking water (20 *μ*g/L) was surpassed in 23 samples and only 5 samples surpassed the WHO guidelines (70 *μ*g/L). As for the other elements (Table 2), private well water source had the most number of samples exceeding the recommended values.

Arsenic (As) is an essential element in ultratrace quantities and higher concentrations of As in drinking water are a worldwide concern. Countries such as Hungary, India, and Bangladesh have coped with severe health problems caused by high concentrations of As in drinking water. It has been documented that As is carcinogen and highly toxic. Chronic poisoning has led to skin lesions and vascular disease [21, 27]. In Kosovo, As concentration levels in drinking water were higher in private wells than in other sources. Maximum acceptable concentration of 10 *μ*g/L (EU and WHO) was exceeded in 29 samples of which 23 were from private wells. 

Aluminum (Al) is considered to be less toxic [21]. Yet, studies have shown that Al in drinking water has been related epidemiologically to Alzheimer's disease [28]. WHO does not have regulated limits for Al in drinking water. The 200 *μ*g/L limit set by the EU for Al in drinking water was exceeded in 36 samples of which 18 were from private wells.

Boron (B) has been recognized as a toxic element and is known to accumulate in the human body and damage the nervous system [21]. The drinking water samples analyzed in this study surpassed the WHO regulated value of 500 *μ*g/L in 20 samples of which 16 came from private wells.

### 3.2. Water Quality

As most of the samples of the four sources were sampled directly from a pipeline system (a common household water distribution system), or other sampling devices (e.g., buckets and electronic pumps), one could argue that contamination occurred from the infrastructure or transport methods. Yet, our goal is to present the elemental analyses using a realistic method of how drinking water is distributed and consumed by the Kosovar population. Furthermore, research has shown that when water is run for at least five minutes before sampling, the water's elemental profile is similar to the natural groundwater structure [29].

The elemental concentration results show that the drinking water is reasonably acceptable with a few exceptions in which some elements were significantly above EU and WHO guidelines. We believe that the high number of drinking water and/or groundwater samples with depth ranging from 1 to 150 meters is a good representation of the quality of water in Kosovo. The surrounding countries of Kosovo lack similar studies and further judgment on the water quality of the region could not be assessed. In the future, our study could be used to evaluate and correlate the contamination caused by different environmental factors.

## 4. Strength and Limitation of the Current Study

We have determined the concentrations of 32 elements in nearly 1,000 water samples from allover Kosovo with ICPMS. A strict QA/QC protocol was employed guaranteeing accurate results. ICPMS is currently the most powerful technique for trace metal analysis, allowing the detection of heavy metals at concentrations as low as 1 ng/L. Our results are very useful to Kosovar authorities for future monitoring studies. Although we found a rather positive situation for most of the elements, we have to admit that our study does not cover all possible contaminates commonly found in water. We determined most of the elements except the halogens. Fluorine, a common contaminant in drinking water, could not be determined with ICPMS because of its high ionization potential. Also, organic pollutants were not the aim of the current study.

## 5. Conclusions

The results of this study should increase the awareness of the importance of drinking water quality in Kosovo as well as enhance the communication of health authorities in Kosovo with the private drinking water source owners. The current condition of the drinking water and/or groundwater in Kosovo can be used by health and governmental authorities to create regulations that set the allowable elemental concentration levels for drinking water. Fortunately, in most of the water samples analyzed, the element concentrations were below the EU and WHO drinking water limits. Epidemiological studies should be followed, along with reported consequences related to higher elemental concentrations in the regions of sampling points in Kosovo. Furthermore, monitoring of seasonal variations of EU-regulated elements is required.

## Figures and Tables

**Table 1:** Values of certified and calculated NIST 1643e SRM reference water analyzed by ICPMS and internal standard used for correction.

	NIST 1643e SRM, *n* = 7			
Element (Unit)	Certified ± STD	Measured ± STD	Trueness mean ± STD	Internal standard
Li (*μ*g/L)	17.4 ± 1.7	17.9 ± 1.0	102.7 ± 5.4	Sc
Be (*μ*g/L)	13.64 ± 0.17	13.50 ± 0.39	99.0 ± 2.9	Sc
B (*μ*g/L)	154 ± 3.9	161.4 ± 7.7	104.8 ± 4.8	Sc
Na (mg/L)	20.23 ± 0.26	20.58 ± 0.42	101.7 ± 2.0	Sc
Mg (*μ*g/L)	7841 ± 98	8077 ± 247	103.0 ± 3.1	Sc
Al (*μ*g/L)	138 ± 8.6	146.0 ± 2.4	105.8 ± 1.7	Sc
K (*μ*g/L)	2034 ± 29	1975 ± 53	97.1 ± 2.7	Sc
Ca (mg/L)	32.3 ± 1.1	30.0 ± 0.89	92.7 ± 3.0	Sc
V (*μ*g/L)	37.86 ± 0.59	37.33 ± 0.60	98.6 ± 1.6	Sc
Cr (*μ*g/L)	20.4 ± 0.24	20.78 ± 0.43	101.8 ± 2.1	Sc
Mn (*μ*g/L)	38.97 ± 0.45	37.73 ± 0.82	96.8 ± 2.2	Sc
Fe (*μ*g/L)	98.1 ± 1.4	100.2 ± 1.1	102.2 ± 1.1	Sc
Co (*μ*g/L)	27.06 ± 0.32	26.66 ± 0.41	98.5 ± 1.5	Sc
Ni (*μ*g/L)	62.41 ± 0.69	61.90 ± 0.78	99.2 ± 1.3	Ge
Cu (*μ*g/L)	22.76 ± 0.31	23.08 ± 1.21	101.4 ± 5.3	Ge
Zn (*μ*g/L)	78.5 ± 2.2	77.37 ± 3.61	98.6 ± 4.7	Ge
As (*μ*g/L)	60.45 ± 0.72	60.90 ± 2.26	100.7 ± 3.7	Ge
Rb (*μ*g/L)	14.14 ± 0.18	13.54 ± 0.47	95.7 ± 3.5	Ge
Sr (*μ*g/L)	323.1 ± 3.6	340.5 ± 20.1	105.4 ± 5.9	Ge
Mo (*μ*g/L)	121.4 ± 1.3	120.8 ± 2.9	99.5 ± 2.4	Ge
Ag (*μ*g/L)	1.062 ± 0.075	1.037 ± 0.067	97.6 ± 6.4	In
Cd (*μ*g/L)	6.568 ± 0.073	6.541 ± 0.191	99.6 ± 2.9	In
Sb (*μ*g/L)	58.3 ± 0.61	50.45 ± 1.69	86.5 ± 3.4	In
Te (*μ*g/L)	1.09 ± 0.11	1.00 ± 0.06	92.1 ± 6.5	In
Ba (*μ*g/L)	544.2 ± 5.8	526.4 ± 18.0	96.7 ± 3.4	In
Tl (*μ*g/L)	7.445 ± 0.096	7.139 ± 0.246	95.9 ± 3.4	Lu
Pb (*μ*g/L)	19.63 ± 0.21	18.94 ± 0.52	96.5 ± 2.8	Lu
Bi (*μ*g/L)	14.09 ± 0.15	13.99 ± 0.49	99.3 ± 3.5	Lu

**Table 2:** Summary of concentrations for the water sources in Kosovo and EU and WHO guidelines.

Type of water	Li *μ*g/L	B *μ*g/L	Na mg/L	Mg mg/L	Al *μ*g/L	K mg/L	Ca mg/L	V *μ*g/L	Cr *μ*g/L	Mn *μ*g/L	Fe *μ*g/L	Co *μ*g/L	Ni *μ*g/L	Cu *μ*g/L	Zn *μ*g/L	As *μ*g/L	Rb *μ*g/L	Sr mg/L	Mo *μ*g/L	Cd *μ*g/L	Sb *μ*g/L	Ba *μ*g/L	Pb *μ*g/L
Artesian (*n* = 201)																							
Mean	7.78	55.8	14.2	19.2	48.1	2.12	65.3	1.40	1.19	31.2	83.5	0.12	4.09	4.08	29.7	1.11	2.55	0.33	0.24	0.038	0.063	48.3	0.95
Minimum	0.019	0.51	0.31	0.36	0.10	0.08	1.08	0.016	0.011	0.014	0.431	0.010	0.045	0.23	0.43	0.023	0.045	0.007	0.011	0.010	0.010	0.41	0.012
25th percentile	0.97	7.06	2.75	5.98	2.52	0.61	36.4	0.17	0.16	0.34	4.24	0.026	0.33	1.03	2.85	0.13	0.28	0.13	0.057	0.012	0.021	10.1	0.076
Median	2.83	12.3	5.63	11.8	7.42	0.96	65.9	0.34	0.33	1.06	11.3	0.044	0.88	1.73	8.37	0.26	0.49	0.24	0.11	0.018	0.034	24.7	0.20
75th percentile	5.40	20.0	12.3	26.5	29.2	1.77	90.0	1.09	0.76	4.45	42.2	0.082	2.27	3.57	27.8	0.62	1.22	0.42	0.23	0.031	0.055	59.2	0.50
Maximum	327.1	5970	509.2	313.0	1052	25.5	171.5	38.5	32.7	2603	3442	3.84	214.5	216.9	413.6	34.0	60.6	2.40	7.34	0.40	0.94	590.5	94.4
Percentage ≤ LOQ	0	5	0	0	6.5	0	0	1	2	7	0	5	9	0	9.5	16.4	0.5	0	10	73.6	10	0	11.4
Private well (*n* = 647)																							
Mean	13.5	100.5	38.4	35.9	47.7	10.5	94.7	1.94	3.47	62.2	128.5	0.22	5.44	5.47	66.8	2.25	3.81	0.60	0.41	0.22	0.14	76.5	0.76
Minimum	0.11	0.33	1.06	2.01	0.069	0.056	10.1	0.042	0.012	0.013	0.58	0.012	0.020	0.24	0.17	0.020	0.029	0.054	0.011	0.010	0.011	2.30	0.010
25th percentile	3.30	22.8	13.8	18.3	2.84	1.53	63.6	0.32	0.29	0.65	7.93	0.06	1.02	1.61	11.9	0.27	0.42	0.30	0.084	0.014	0.039	38.7	0.078
Median	7.97	50.5	28.0	31.2	7.95	3.27	88.6	0.64	0.70	1.75	17.2	0.092	2.02	2.67	25.9	0.63	0.91	0.49	0.17	0.022	0.066	61.0	0.16
75th percentile	16.3	119.8	52.9	47.4	20.8	8.79	115.6	1.57	2.11	6.21	43.6	0.16	4.47	4.89	54.9	2.06	2.71	0.73	0.35	0.038	0.13	100.6	0.39
Maximum	207.0	1844	259.1	193.8	5383	188.1	410.2	45.5	760.1	15580	19940	27.3	901.6	135.3	5610	61.4	88.5	4.20	9.35	34.0	4.57	352.3	96.3
Percentage ≤ LOQ	0	0.5	0	0	3.1	0	0	0	0.6	1.7	0	0.5	8.2	0	5.4	19.8	0	0	1.4	64.6	0.9	0	3.1
Public water (*n* = 33)																							
Mean	5.63	45.1	11.7	17.1	93.7	1.56	61.0	0.41	0.89	13.5	51.0	0.09	1.68	4.17	68.7	0.79	0.57	0.24	0.14	0.030	0.12	31.2	0.32
Minimum	0.11	0.42	0.78	1.50	0.058	0.30	6.88	0.022	0.024	0.052	0.42	0.010	0.13	0.51	0.66	0.055	0.10	0.041	0.015	0.011	0.010	2.60	0.016
25th percentile	0.96	5.74	1.72	4.26	7.63	0.79	37.0	0.19	0.20	0.66	6.14	0.027	0.45	1.45	5.55	0.19	0.28	0.14	0.052	0.013	0.023	11.5	0.066
Median	1.49	16.8	3.71	8.64	30.3	0.97	51.9	0.28	0.47	2.96	12.7	0.043	0.88	2.39	11.0	0.45	0.46	0.19	0.10	0.017	0.046	19.7	0.21
75th percentile	3.17	45.6	8.21	16.9	103.0	1.88	81.6	0.54	1.15	6.84	39.2	0.091	3.03	4.81	27.9	0.75	0.93	0.36	0.18	0.041	0.075	45.8	0.30
Maximum	48.0	416.1	68.8	107.7	621.4	5.12	134.3	1.43	6.33	226.7	488.0	0.79	6.10	34.8	973.2	7.20	1.62	0.76	0.62	0.094	1.79	96.5	2.13
Percentage ≤ LOQ	0	6.1	0	0	6.1	0	0	0	0	3	0	0	15.2	0.0	15.2	15.2	0	0	3	78.8	3	0	3
Pumped (*n* = 70)																							
Mean	15.7	147.3	37.8	33.5	52.1	8.41	87.6	2.12	2.07	95.2	258.0	0.42	5.60	6.69	63.1	2.41	5.01	0.54	0.72	0.074	0.10	76.0	0.79
Minimum	0.28	4.30	1.75	4.68	0.073	0.27	1.33	0.013	0.043	0.033	0.68	0.016	0.082	0.041	1.14	0.041	0.057	0.012	0.018	0.011	0.012	1.13	0.010
25th percentile	3.84	20.6	13.0	15.3	1.65	1.26	62.1	0.25	0.29	1.02	8.31	0.058	0.94	1.63	10.5	0.26	0.39	0.35	0.077	0.017	0.026	40.9	0.14
Median	8.42	45.2	26.1	30.3	7.21	2.02	87.1	0.48	0.77	6.74	30.3	0.12	1.93	3.81	24.6	0.57	0.92	0.47	0.17	0.022	0.048	67.3	0.28
75th percentile	15.0	103.1	37.3	50.2	19.0	5.68	105.7	1.37	2.90	29.0	140.1	0.24	4.93	6.13	50.1	1.54	1.88	0.68	0.44	0.054	0.10	102.4	0.63
Maximum	215.3	4983	483.5	93.1	1360	197.6	236.0	22.8	26.7	2842	4052	6.21	83.5	53.4	610.8	44.2	88.5	2.46	16.1	1.02	1.84	229.8	16.6
Percentage ≤ LOQ	0	1.4	0	0	2.9	0	0	0	2.9	8.6	0	2.9	1.4	0	2.9	8.6	0	0	0	55.7	5.7	1.4	2.9
EU guidelines (1998)		1000	200		200				50	50	200		20	2000		10			70	5	5		10
WHO guidelines (2003)		500							50	400			70	2000		10				3	20	700	10

**Table 3:** Total samples exceeding the EU and WHO drinking water guidelines.

Element	EU					WHO				
	Artesian	Private well	Public water	Pumped	Total	Artesian	Private well	Public water	Pumped	Total
B	1	4	—	1	6	3	16	—	1	20
Na	1	2	—	1	4	—	—	—	—	—
Al	10	18	3	5	36	—	—	—	—	—
Cr	—	2	—	—	2	—	2	—	—	2
Mn	13	49	1	11	74	3	15	—	2	20
Fe	15	32	2	13	62	—	—	—	—	—
Ni	5	16	—	2	23	2	2	—	2	20
Cu	—	—	—	—	—	—	—	—	—	—
As	2	23	—	4	29	2	23	—	4	29
Mo	—	—	—	—	—	—	—	—	—	—
Cd	—	2	—	—	2	—	2	—	—	2
Sb	—	—	—	—	—	—	—	—	—	—
Ba	—	—	—	—	—	—	—	—	—	—
Pb	2	4	—	1	7	2	4	—	1	7
U	—	—	—	—	—	6	61	—	2	69
